# Mineral and Bone Disease in African Renal Failure Patients

**DOI:** 10.5812/numonthly.9476

**Published:** 2013-03-30

**Authors:** Kaan Savas Gulleroglu, Nadide Basak Gulleroglu

**Affiliations:** 1 Pediatric Nephrology Division, Adana Numune Training and Research Hospital, Adana, Turkey; 2 Radiology Division, Adana Numune Training and Research Hospital, Adana, Turkey

**Keywords:** Renal Insufficiency, Chronic, Hyperparathyroidism

Dear Editor,

“Mineral and bone disease in black African hemodialysis patients: a report from senegal” by Seck and all published in Nephro-Urology Monthly is an interesting paper (1).

Disturbances in mineral and bone metabolism are common in patients with chronic kidney disease (CKD). Patients with CKD almost always develop secondary hyperplasia of the parathyroid glands, resulting in elevated blood levels of parathyroid hormone (PTH) (2). Normal parathyroid cells are characterized by an extremely low turnover. In chronic renal failure, the increase in parathyroid mass is mainly due to enhanced cell proliferation, not cell hypertrophy. Slow development of hyperplasia of the parathyroid glands could explain the slow evolution of secondary hyperparathyroidism in patients on dialysis for a long time. Parathyroid gland mass progressively grows with time on long-term dialysis treatment (3). Despite a long median time of dialysis duration as 45 ± 30.5 months, Seck and all could not find any association between CKD related mineral bone disease (CKD-MBD) and dialysis duration. They also could not show any relation between subtype of CKD-MBD, age, gender, dialysis effectiveness. In K/DOQI: clinical practice guidelines for bone metabolism and disease in chronic kidney disease, possible reasons for variations in CKD-MBD are age of patient, genetic effects, type of underlying kidney disease, duration of kidney failure, relative severity of the pathogenetic processes underlying the derangement in bone metabolism, differences in dietary habits, type of therapy used, treatment with dialysis and its duration, aluminum burden and diabetes (2).

Bone radiographs are not indicated for the assessment of bone disease of CKD. Seck and all used X-ray for detecting looser’s zones and vascular calcifications. Bone radiographs are also useful in detecting calcification articular cartilages and periarticular tissues in renal osteodistrophy. On the other hand, osteosclerosis occurs in 9-34% of patients (4).

CKD-MBD is a common worldwide problem in patients with CKD. Adequate dialysis and medical treatment are required for the limitation of the destruction of the disease. More effort is needed in this area.
